# Anatomical harmonics basis based brain source localization with application to epilepsy

**DOI:** 10.1038/s41598-022-14500-7

**Published:** 2022-07-04

**Authors:** Amita Giri, Lalan Kumar, Nilesh Kurwale, Tapan K. Gandhi

**Affiliations:** 1 Department of Electrical Engineering, Indian Institute of Technology - Delhi, New Delhi, India; 2 Department of Electrical Engineering and Bharti School of Telecommunication, Indian Institute of Technology - Delhi, New Delhi, India; 3 Deenanath Mangeshkar Hospital and Research Center, Pune, Maharashtra India

**Keywords:** Biomedical engineering, Brain imaging

## Abstract

Brain Source Localization (BSL) using Electroencephalogram (EEG) has been a useful noninvasive modality for the diagnosis of epileptogenic zones, study of evoked related potentials, and brain disorders. The inverse solution of BSL is limited by high computational cost and localization error. The performance is additionally limited by head shape assumption and the corresponding harmonics basis function. In this work, an anatomical harmonics basis (Spherical Harmonics (SH), and more particularly Head Harmonics (H^2^)) based BSL is presented. The spatio-temporal four shell head model is formulated in SH and H^2^ domain. The anatomical harmonics domain formulation leads to dimensionality reduction and increased contribution of source eigenvalues, resulting in decreased computation and increased accuracy respectively. The performance of spatial subspace based Multiple Signal Classification (MUSIC) and Recursively Applied and Projected (RAP)-MUSIC method is compared with the proposed SH and H^2^ counterparts on simulated data. SH and H^2^ domain processing effectively resolves the problem of high computational cost without sacrificing the inverse source localization accuracy. The proposed H^2^ MUSIC was additionally validated for epileptogenic zone localization on clinical EEG data. The proposed framework offers an effective solution to clinicians in automated and time efficient seizure localization.

## Introduction

The use of an accurate and efficient algorithm for Brain Source Localization (BSL) using Electroencephalography (EEG) measurements has been used in various neuroscience applications such as pre-surgical mapping in patients undergoing resection of epileptogenic zones^1–4^, Brain Computer Interface (BCI) based prosthetic limbs^5,6^, and attention deficit hyperactive disorder^7,8^. Among these diseases, epilepsy is the most important and common neurological disorder as $1\%$ of the world population is suffering from it. To solve this problem, EEG is regarded as the most commonly used non-invasive diagnosis tool due to its high temporal resolution, portability and cost-effectiveness. The process of brain source localization is carried out in two phases, which are forward modeling and inverse modeling.

Forward modeling involves estimation of the scalp potentials, given head model and current source. The volume conductor head model is chosen based on application, accuracy and computational complexity. The two kinds of volume conductor head models include numerical and analytical model. The numerical models are based on realistic modeling of human head with more complexity and low localization error. Boundary Element Method (BEM)^9^, Finite Element Method (FEM)^10^, and Finite Difference Method (FDM)^11^ belong to numerical model category. In analytical modeling, head is approximated as a set of concentric spheres, each with homogeneous conductivity. This approximation results in low computational complexity. In literature, a single-shell head model^12^ with a homogeneous conductivity was reported first. Later, three-shell^13^ volume conductor head model was introduced where conductivity of skull was found to be lower than that of scalp/brain tissues. A more accurate four shell head model^14^ with additional CerebroSpinal Fluid (CSF) layer between brain and skull, was introduced thereafter that forms the basis of the current work.

The recorded scalp potentials are further utilized to solve the inverse problem for active source localization. BSL methods are widely classified into distributed source (dipole-imaging) model and the dipole-fitting model. The distributed source model assumes that there is a large number of sources confined in an active region of the brain. The BSL, in this case, refers to the estimation of active source amplitudes and orientations using linear optimization techniques such as Bayesian methods, Minimum Norm Estimates (MNE), Weighted Minimum Norm Estimates (WMNE), Low Resolution Electrical Tomography (LORETA), and Local Auto Regressive Average (LAURA). The distributed source model based BSL being highly underdetermined, requires additional constraints like least energy, smooth variation and sparse focal nature of the sources are required to obtain better source estimation^15^. However, the localization of a limited number of equivalent dipoles is the most classical approach to solve the inverse problem. The a priori assumption in this solution is that only one or a few active areas in the brain generated the scalp potential field. Under this constraint, non-linear multidimensional optimization procedures allow to determine the dipole parameters that best explain the observed scalp potential measurements. This includes subspace based Multiple Signal Classification (MUSIC)^16^, Recursively Applied and Projected (RAP) MUSIC^17^, Truncated RAP-MUSIC (TRAP-MUSIC)^18^), beamforming, and genetic algorithms.

In the process of source localization, the inverse solutions is evaluated at each grid point i.e. nearly thousands of times to obtain convergent results. Practical computations of these inverse solutions are considerably expensive. The high computational cost involved thus becomes one of the major obstacles in solving the inverse problem of the EEG. In this work, we proposed a novel framework for active brain source localization by utilizing anatomical harmonics (Spherical Harmonics (SH) and Head Harmonics (H^2^)) domain processing on measured EEG data. SH have been widely used for acoustic source localization due to ease of array processing in SH domain^19,20^. It requires sensor array to be arranged on a rigid spherical baffle. In literature, the human head is approximated by spherical shape^12–14^. Hence, SH basis functions have been natural choice for accurate representation of EEG data over head^21,22^. Spherical harmonics were utilized in^21^ for source reconstruction using dipole imaging method. Surface complexity of brain is estimated in^22^ using SH to differentiate autistics, dyslexics, and controls subjects. The inherent assumption in application of SH basis function is that the data is present over entire sphere. However, the cap where the sensors are placed for data acquisition, assumes the shape between hemisphere and sphere. Hence, a more realistic basis function, Head Harmonics (H$^2$) were utilized in^23^ for Infinite Homogeneous Isotropic Conductor (IHIC) head model and in^24^ for Three Layer Concentric Spherical (TLCS) head model. In this work, more realistic four layer head model is first formulated in SH domain followed by H^2^ domain. The transformed model is then used for BSL using the dipole fitting approach. In particular, MUSIC and RAP-MUSIC BSL methods are presented in SH and H^2^ domain.

## Four shell based EEG forward model in spatio-temporal domain

**Figure 1:**
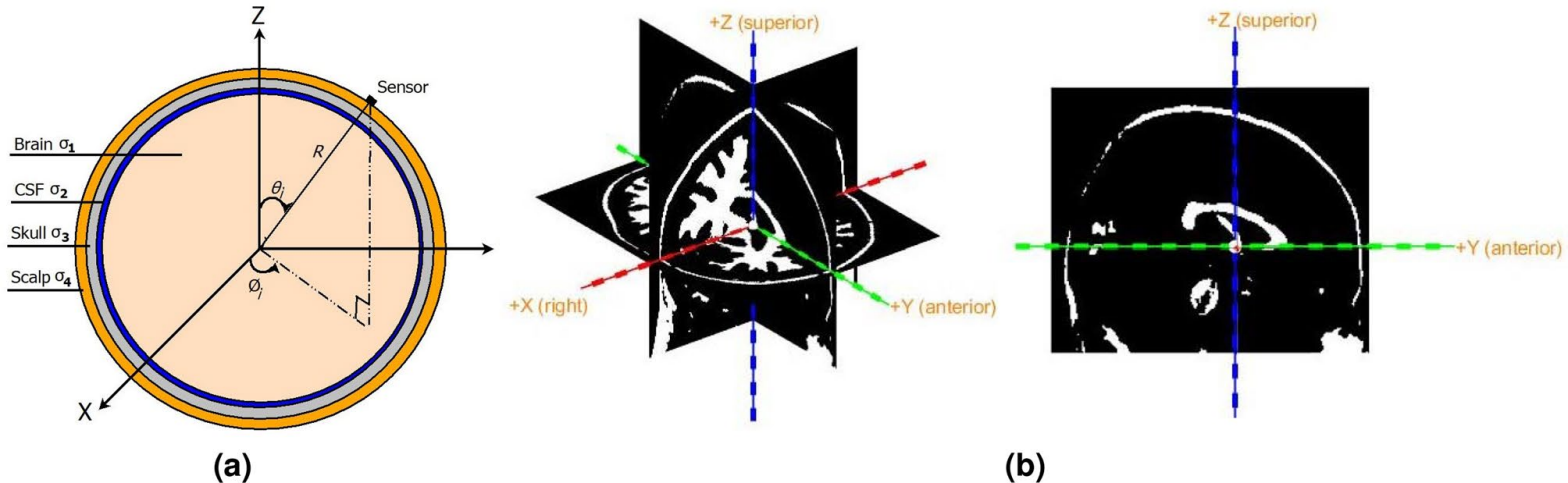
(**a**) Four shell head model, and (**b**) coordinate system.

Spatio-temporal domain data model accounts for change in EEG potential w.r.t. space and time. Forward model is combination of head model and source model. The head model utilized herein is four layer concentric shell head model as shown in Fig. 1a, with the coordinate system in Fig. 1b. Brain, CSF, skull and scalp constitute the four concentric spherical layers with conductivity as $\sigma _1$, $\sigma _2$, $\sigma _3$ and $\sigma _4$ respectively. The brain comprises of homogeneous neural tissues and forms the innermost concentric sphere of radius *br*. This is surrounded by a concentric outer spherical shell of radius *cr* representing the CSF. This is followed by a third layer with radius *dr* for the skull. The outermost layer is the scalp of radius *R*. The BSL problem under consideration, utilizes Equivalent Current Dipole (ECD) source model where each dipole is parameterized by its location ${\mathbf {{r}}_p}$ and dipole moment ${\mathbf {m}}_p$.

*I* Sensors are placed over the scalp to record brain electrical activity due to *P* active dipole sources. The location of the *i*th sensor and *p*th source is given by

1 $$\begin{aligned} {\mathbf {\textit{r}}}_{\mathbf {\textit{i}}} = (R,\Omega _i) = (R, \theta _i,\phi _i) = (x_i,y_i,z_i),~~~~ \text {and}~~~~ {\mathbf {{r}}_p} = (r_p,\Omega _p) = (r_p, \theta _p,\phi _p) = (x_p,y_p,z_p) \end{aligned}$$

respectively, where $\theta$ is elevation angle measured downward from positive *Z* axis, and $\phi$ is azimuth angle measured anticlockwise from positive *X* axis. All the dipole sources are assumed to be inside the brain compartment of the four shell model. The EEG potential $V({\mathbf {\textit{r}}}_{\mathbf {\textit{i}}},{\mathbf {{r}}_p},t)$ measured at *i*th sensor location generated by *p*th active source dipole at a time instant *t* is given by^14^

2 $$\begin{aligned} V({\mathbf {\textit{r}}}_{\mathbf {\textit{i}}},{\mathbf {{r}}_p},t)&= \frac{1}{4\pi \sigma _4 R^2} {{\sum }}_{n=1}^{\infty } \Bigg \{c_n \begin{bmatrix}{f_{n}}({\mathbf {{r}}_p}) \end{bmatrix} {\mathbf {m}}_p(t) \cdot \begin{bmatrix}\mathbf {r_o} P_n(\cos ~\Theta ) ~+~ \mathbf {t_o} \frac{{P^{1}_n}(\cos ~\Theta ) }{n}\end{bmatrix}\Bigg \} , \nonumber \\&\quad \text {where}~~~c_n = \frac{(2n+1)^4 (cd)^{2n+1}}{\kappa },\nonumber \\ \kappa&= d^{2n+1} \Bigg \{b^{2n+1}\left( \frac{\sigma _1}{\sigma _2}-1\right) \left( \frac{\sigma _2}{\sigma _3}-1\right) (n+1) +c^{2n+1}\left( \frac{\sigma _1}{\sigma _2}n+n+1\right) \left( \frac{\sigma _2}{\sigma _3}n+n+1\right) \Bigg \} \nonumber \\&\quad \Bigg \{\left( \frac{\sigma _3}{\sigma _4}n+n+1\right) + (n+1)\left( \frac{\sigma _3}{\sigma _4}-1\right) d^{2n+1}\Bigg \}\nonumber \\&\quad +(n+1)c^{2n+1}\Bigg \{\{b^{2n+1}\left( \frac{\sigma _1}{\sigma _2} -1\right) \left( \frac{\sigma _2}{\sigma _3}n+\frac{\sigma _2}{\sigma _3}+n\right) \nonumber \\&\quad c^{2n+1}\left( \frac{\sigma _1}{\sigma _2}n+n+1\right) \left( \frac{\sigma _2}{\sigma _3}-1\right) \Bigg \} \Bigg \{n\left( \frac{\sigma _3}{\sigma _4}-1\right) +\left( \frac{\sigma _3}{\sigma _4}n+\frac{\sigma _3}{\sigma _4}+n\right) d^{2n+1} \Bigg \} \end{aligned}$$

and $(\cdot )$ represents the vector dot product. Here $\{b,c, d\} < 1$ denote the relative radii of brain, CSF and skull with respect to the scalp radius. Eccentricity of the dipole is ${r}_p/R$ and $$\begin{bmatrix}{f_{n}}({\mathbf {{r}}_p})\end{bmatrix}=({r}_p/R)^{n-1}$$. Radial unit vector is given by $\mathbf {r_o}={\mathbf {{r}}_p}/ |{\mathbf {{r}}_p}|$ and $\mathbf {t_o}$ represents tangential unit vector defined in terms of vector cross product as

3 $$\begin{aligned} \mathbf {t_o} = \frac{{\mathbf {{r}}_p}\times {\mathbf {\textit{r}}}_{\mathbf {\textit{i}}}\times {\mathbf {{r}}_p}}{\mathbf {\mid } {\mathbf {{r}}_p}\times {\mathbf {\textit{r}}}_{\mathbf {\textit{i}}}\times {\mathbf {{r}}_p} \mathbf {\mid }} \end{aligned}$$

${P_n}(\cos ~\Theta )$ is the legendre polynomial and ${P^{1}_n}(\cos ~\Theta )$ is the associated legendre polynomial of order $n \in \{1,\ldots ,\infty \}$. The terms corresponding to $n=0$ have zero contribution in (2) and is therefore not considered. $\Theta$ is the angle between *i*th sensor and *p*th dipole with $\cos \Theta$ given as

4 $$\begin{aligned} \cos ~\Theta = \cos \theta _i \cos \theta _p~+~ \cos (\phi _i - \phi _p)\sin \theta _i \sin ~\theta _p \end{aligned}$$

Under the assumption of fixed dipole source location and orientation, dipole moment vector ${\mathbf {m}}_p(t)$ can be written as ${\mathbf {m}}_p(t) = {\mathbf {e}}_ps_p(t)$^16^, where ${\mathbf {e}}_p = [{e}_{px}~{e}_{py}~{e}_{pz}]^{T}$ is unit orientation vector and $s_p(t)$ is magnitude of the dipole moment. Substituting ${\mathbf {m}}_p(t)$ in (2) and rearranging, the EEG potential at *i*th sensor due to *p*th dipole source can now be written as

5 $$\begin{aligned} \begin{aligned} V({\mathbf {\textit{r}}}_{\mathbf {\textit{i}}},{\mathbf {{r}}_p},t) = \frac{1}{4\pi \sigma _4 R^2} {{\sum }}_{n=1}^{\infty }\Bigg \{ c_n \begin{bmatrix}{f_{n}}({\mathbf {{r}}_p})\end{bmatrix}\begin{bmatrix} \mathbf {r_o} P_n(\cos ~\Theta ) ~+~ \mathbf {t_o} \frac{{P^{1}_n}(\cos ~\Theta )}{n}\end{bmatrix}\Bigg \}\cdot {\mathbf {e}}_ps_p(t) \end{aligned} \end{aligned}$$

From (5), the total EEG potential at the *i*th electrode due to all the *P* active dipoles can be written as

6 $$\begin{aligned} V({\mathbf {\textit{r}}}_{\mathbf {\textit{i}}},t) = \sum _{p=1}^{P} {\mathbf {g}}({\mathbf {\textit{r}}}_{\mathbf {\textit{i}}},{\mathbf {{r}}_p})\cdot {\mathbf {e}}_p s_p(t) \end{aligned}$$

where ${\mathbf {g}}({\mathbf {\textit{r}}}_{\mathbf {\textit{i}}},{\mathbf {{r}}_p})_{1 \times 3}$ is gain vector, expressed as

7 $$\begin{aligned} \begin{aligned} {\mathbf {g}}({\mathbf {\textit{r}}}_{\mathbf {\textit{i}}},{\mathbf {{r}}_p}) = \frac{1}{4\pi \sigma _4 R^2} {{\sum }}_{n=1}^{\infty } \Bigg \{c_n \begin{bmatrix}{f_{n}}({\mathbf {{r}}_p})\end{bmatrix} \begin{bmatrix} \mathbf {r_o} P_n(\cos ~\Theta ) ~+~ \mathbf {t_o} \frac{{P^{1}_n}(\cos ~\Theta )}{n}\end{bmatrix}\Bigg \}, \end{aligned} \end{aligned}$$

for four shell model. For *I* electrodes and *P* dipoles, the EEG potential in (6), can be written in matrix form as

8 $$\begin{aligned} \begin{bmatrix} V({\mathbf {\textit{r}}}_1,t)\\ V({\mathbf {\textit{r}}}_2,t) \\ \vdots \\ V({\mathbf {\textit{r}}}_{\mathbf {\textit{I}}},t) \end{bmatrix} = \begin{bmatrix} \mathbf {g^{T}}({\mathbf {\textit{r}}}_1,{\mathbf {r}}_{1}) &{} \dots &{} \mathbf {g^{T}}({\mathbf {\textit{r}}}_1,{\mathbf {r}}_P) \\ \mathbf {g^{T}}({\mathbf {\textit{r}}}_2,{\mathbf {r}}_{1}) &{} \dots &{} \mathbf {g^{T}}({\mathbf {\textit{r}}}_2,{\mathbf {r}}_P) \\ \vdots &{} \ddots &{} \vdots \\ \mathbf {g^{T}}({\mathbf {\textit{r}}}_{\mathbf {\textit{I}}},{\mathbf {r}}_{1}) &{} \dots &{} \mathbf {g^{T}}({\mathbf {\textit{r}}}_{\mathbf {\textit{I}}},{\mathbf {r}}_P) \\ \end{bmatrix} \begin{bmatrix} {\mathbf {M}} \end{bmatrix} \begin{bmatrix} s_{1}(t) \\ s_{2}(t) \\ \vdots \\ s_{P}(t) \end{bmatrix} \end{aligned}$$

Re-writing (8) in compact form, we have

9 $$\begin{aligned} 
\begin{bmatrix} {{V}{(t)}} \end{bmatrix}_{I \times 1}&= \begin{bmatrix} {\mathbf {G}}\end{bmatrix}_{I \times 3P} \begin{bmatrix}{\mathbf {M}}\end{bmatrix}_{3P \times P} \begin{bmatrix}\mathbf {{s}}(t)\end{bmatrix}_{P \times 1} \\ \begin{bmatrix} {\mathbf {G}}\end{bmatrix}&= \begin{bmatrix} G({\mathbf {r}}_{1})&G({\mathbf {r}}_{2})&\cdots&G({\mathbf {r}}_P) \end{bmatrix} \end{aligned}$$

10 $$\begin{aligned} \begin{bmatrix} {\mathbf {M}} \end{bmatrix}&= \text {diag}({\mathbf {e}}_{1},{\mathbf {e}}_{2},\cdots ,{\mathbf {e}}_{P}) \end{aligned}$$

where elements of $G({\mathbf {r}}_p)$ is given by (7), and $[\mathbf{M }]$ is referred as orientation moment matrix. In the presence of spatially and temporally zero mean white Gaussian noise ${{\mathbf {Z}}}$ with variance $\sigma ^2$, the four shell spatio-temporal data model in (9) for $N_s$ time snapshots can be written as

11 $$\begin{aligned} \begin{bmatrix} {\mathbf {V}} \end{bmatrix}_{I \times N_S} = \begin{bmatrix} {\mathbf {A}} \end{bmatrix}_{I \times P} \begin{bmatrix} {\mathbf {S}} \end{bmatrix}_{P \times N_S} + \begin{bmatrix} {\mathbf {Z}} \end{bmatrix}_{I \times N_S} \end{aligned}$$

where manifold matrix $$\begin{bmatrix} {\mathbf {A}} \end{bmatrix} = \begin{bmatrix} {\mathbf {G}} \end{bmatrix}\begin{bmatrix} {\mathbf {M}} \end{bmatrix}$$ is collection of manifold vectors given by

12 $$\begin{aligned} {\mathbf {a}} ({\mathbf {r}}_p) = \begin{bmatrix} {a} ({\mathbf {\textit{r}}}_1,{\mathbf {r}}_p)&{a} ({\mathbf {\textit{r}}}_2,{\mathbf {r}}_p)&\cdots&{a} ({\mathbf {\textit{r}}}_I,{\mathbf {r}}_p) \end{bmatrix}^{T} = \begin{bmatrix} \mathbf {g^{T}}({\mathbf {\textit{r}}}_1,{\mathbf {r}}_{p}){\mathbf {e}}_p&\mathbf {g^{T}}({\mathbf {\textit{r}}}_2,{\mathbf {r}}_{p}){\mathbf {e}}_p&\cdots&\mathbf {g^{T}}({\mathbf {\textit{r}}}_{\mathbf {\textit{I}}},{\mathbf {r}}_{p}){\mathbf {e}}_p \end{bmatrix}^{T} = G({\mathbf {r}}_p){\mathbf {e}}_p \end{aligned}$$

It is to be noted that matrix ${\mathbf {S}}$ is order independent and hence would remain same in the order limited scenario.

## Manifold matrix

In this Section, the spatial domain manifold matrix is expressed in terms of spherical harmonics basis functions ($Y^{m}_{n}$) corresponding to sensor and source coordinates. Spherical harmonic addition theorem suggests^25^

13 $$\begin{aligned} P_n(\cos {\Theta }) = \frac{4\pi }{2n+1} \sum _{m=-n}^{+n} Y^{m}_{n}(\Omega _i)~Y^{m}_{n}(\Omega _p) \end{aligned}$$

Substituting (14) in the gain vector ${\mathbf {g}}({\mathbf {\textit{r}}}_{\mathbf {\textit{i}}},{\mathbf {{r}}_p})$ in (7), we have

14 $$\begin{aligned} \begin{aligned} {\mathbf {g}}({\mathbf {\textit{r}}}_{\mathbf {\textit{i}}},{\mathbf {{r}}_p}) = \frac{1}{\sigma _4 R^2} \sum _{n=1}^{\infty } \frac{c_n \begin{bmatrix}{f_{n}}({\mathbf {{r}}_p})\end{bmatrix}}{2n+1}\begin{bmatrix} \mathbf {r_o} + \alpha _n\mathbf {t_o} \end{bmatrix} \sum _{m=-n}^{+n} Y^{m}_{n}(\Omega _i)~Y^{m}_{n}(\Omega _p),~~~~~~\text {where}~~~~~~~\alpha _n = \frac{P^{1}_n(\cos ~\Theta )}{n P_n(\cos ~\Theta )} \end{aligned} \end{aligned}$$

Radial and tangential orientation component of a dipole are expressed as

15 $$\begin{aligned} e_{pr} = \mathbf {r_o} \cdot {\mathbf {e}}_p ~~~~ \text {and} ~~~~ e_{pt} = \mathbf {t_o} \cdot {\mathbf {e}}_p \end{aligned}$$

respectively. For a radially oriented dipole, $e_{pr} = 1$ and $e_{pt} = 0$. For a tangential oriented dipole $e_{pt} = 1$ and $e_{pr} = 0$. A mixed oriented dipole has both radial and tangential component as non-zero. As the EEG reflects the electrical activity of the post-synaptic potentials of pyramidal neuron cells oriented perpendicular to the cortical surface, dipoles with radial orientation is considered herein. For a radially oriented dipole, the element ${a}({\mathbf {\textit{r}}}_{\mathbf {\textit{i}}},{\mathbf {{r}}_p})=\mathbf {g^{T}}({\mathbf {\textit{r}}}_i,{\mathbf {r}}_{p}){\mathbf {e}}_p$ of a manifold vector can be written utilizing (15) and (16) as

16 $$\begin{aligned} \begin{aligned} {a}({\mathbf {\textit{r}}}_{\mathbf {\textit{i}}},{\mathbf {{r}}_p}) =&\sum _{n=1}^{\infty } b_n({\mathbf {{r}}_p}) \Bigg \{\sum _{m=-n}^{+n} Y^{m}_{n}(\Omega _i)~Y^{m}_{n}(\Omega _p) \Bigg \} \end{aligned},~~~\text {with}~~~~~b_n({\mathbf {{r}}_p}) = \frac{c_n\begin{bmatrix}f_n({\mathbf {{r}}_p})\end{bmatrix}}{\sigma _4 R^2(2n+1)} \end{aligned}$$

**Figure 2:**
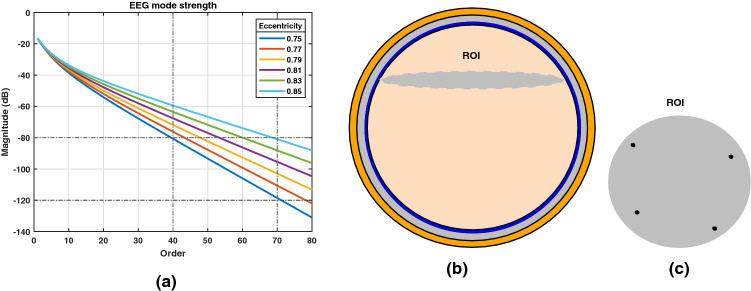
(**a**) EEG mode strength $b_n({\mathbf {{r}}_p})$ with order *n* of different source eccentricity. (**b**) The 2-D axial termed as ROI was taken at the depth of 3.2 *cm* from the top of the scalp surface. (**c**) Four active sources were placed pseudo-randomly on the ROI.

The expression in (17), can not be computed in practice due to the infinite series summation. EEG mode strength $b_n({\mathbf {{r}}_p})$ determines the relative weighting of sensor and source spherical harmonics with the EEG signal order *n*. The parameter $b_n({\mathbf {{r}}_p})$ is a function of head model and the source radial position and is plotted in Fig. 2(a). The magnitude of $b_n({\mathbf {{r}}_p})$ diminishes significantly after order 40 and therefore the EEG mode strength becomes insignificant for higher order. This suggests that the summation in (17) can be truncated to a finite order $N_{ref}$ upto $40-70$ without any significant error.

Under the assumption of finite order, *n* takes value from $\{1,\ldots ,N_{ref}\}$, where $N_{ref}$ will be referred as EEG signal order. For a given order *n*, the degree *m* varies from $\{-n,\ldots ,n\}$. Thus, there are total $\Lambda _{ref} = (N_{ref}+1)^2-1$ distinct spherical harmonics $Y_{n}^{m}$. Hence, rewriting (17) in the matrix form,

17 $$\begin{aligned} \begin{aligned} {a}({\mathbf {\textit{r}}}_{\mathbf {\textit{i}}},{\mathbf {{r}}_p}) = \begin{bmatrix}{\mathbf {Y}}(\Omega _i) \end{bmatrix}_{1 \times \Lambda _{ref}} \begin{bmatrix}{\mathbf {B}}({\mathbf {{r}}_p}) \end{bmatrix}_{\Lambda _{ref} \times \Lambda _{ref}}\begin{bmatrix}{\mathbf {Y}}(\Omega _p)^{T} \end{bmatrix}_{\Lambda _{ref} \times 1} \end{aligned} \end{aligned}$$

where ${\mathbf {Y}}(\Omega _i)$ is defined as

18 $$\begin{aligned} {\mathbf {Y}}(\Omega _i) = \begin{bmatrix} Y^{-1}_{1}(\Omega _i)~~Y^{0}_{1}(\Omega _i)~~Y^{1}_{1}(\Omega _i)\cdots Y^{N_{ref}}_{N_{ref}}(\Omega _i)\end{bmatrix} \end{aligned}$$

${\mathbf {Y}}(\Omega _p)$ is defined by replacing the argument to $\Omega _p$ in (19). EEG mode strength ${\mathbf {B}}({\mathbf {{r}}_p})$ is a diagonal matrix given by

19 $$\begin{aligned} {\mathbf {B}}({\mathbf {{r}}_p}) = diag(b_1({\mathbf {{r}}_p}),~b_1({\mathbf {{r}}_p}),~b_1({\mathbf {{r}}_p}),~\ldots ,~b_{N_{ref}}({\mathbf {{r}}_p})) \end{aligned}$$

The manifold matrix $$\begin{bmatrix} {\mathbf {A}}\end{bmatrix}_{I \times P}$$ for a total of *I* electrodes and *P* dipoles can now be written as

20 $$\begin{aligned} \begin{bmatrix} {\mathbf {A}}\end{bmatrix}_{I \times P} =&\begin{bmatrix}{\mathbf {Y}}(\Phi ) \end{bmatrix}_{I \times \Lambda _{ref}} \begin{bmatrix}{\mathbf{B}} \end{bmatrix}_{\Lambda _{ref} \times \Lambda _{ref} P}\begin{bmatrix}{\mathbf {Y}}(\Psi ) \end{bmatrix}_{\Lambda _{ref} P \times P}, ~~\text {where} \end{aligned}$$

21 $$\begin{aligned} {\mathbf {Y}}(\Phi )&= \begin{bmatrix} {\mathbf {Y}}(\Omega _1)^{T}&{\mathbf {Y}}(\Omega _2)^{T}&\cdots&{\mathbf {Y}}(\Omega _I)^{T}\end{bmatrix}^{T}_{I \times \Lambda _{ref}} \end{aligned}$$

22 $$\begin{aligned} {\mathbf{B}}&= \begin{bmatrix} {{\mathbf {B}}({\mathbf {r}}_1)} ~~ {\mathbf{B}({\mathbf {r}}_2)} ~~ \cdots ~~ {{\mathbf {B}}({\mathbf {{r}}_p})} \end{bmatrix}_{\Lambda _{ref} \times \Lambda _{ref} P} \end{aligned}$$

23 $$\begin{aligned} \begin{bmatrix}{\mathbf {Y}}(\Psi )_{\Lambda _{ref} P \times P} \end{bmatrix}&= \begin{bmatrix} {\mathbf {Y}}{(\Omega _1)}^{T} &{} &{} {\text {\huge 0}}\\ &{} {\mathbf {Y}}{(\Omega _2)}^{T} &{} \\ &{} {\text {\huge 0}}&{} \ddots \\ &{} &{} &{} {\mathbf {Y}}{(\Omega _P)}^{T} \\ \end{bmatrix}_{\Lambda _{ref} P \times P} \end{aligned}$$

and $\Phi$, $\Psi$ contain all sensors and dipoles position ($\theta$,$\phi$)’s respectively. It may be noted from (21) that the manifold matrix is the product of SH basis functions corresponding to sensor coordinates, EEG mode strength and the SH basis functions corresponding to source coordinates.

## Four shell based EEG forward model in spherical harmonics domain

In this Section, four shell based EEG forward model is presented in spherical harmonics domain. The spherical harmonic basis functions is first introduced followed by a detailed derivation of EEG signal decomposition in SH domain.

### The spherical harmonics basis functions

The head model considered herein is four shell spherical model. Hence, the SH basis functions becomes a natural choice to a function defined on the head surface. In this work, SH basis functions are explored for forward problem formulation and subsequent localization, followed by a more accurate head harmonics^23,24^ basis functions based approach.

As EEG signals are real and discrete in nature, the real Spherical Fourier Transform (SFT) is applied instead of complex SFT. The real SFT pair for the discrete time EEG signal is given by^25^

24 $$\begin{aligned} V_{nm} = \int _{0}^{2\pi } \int _{0}^{\pi }V(\theta ,\phi )[Y_{n}^m(\theta ,\phi )]\sin \theta d\theta d\phi ,~~~~~~~~~~~ \longleftrightarrow ~~~~~~~~~~~~~~ V(\theta ,\phi ) = \sum _{n=0}^{N_{a}}\sum _{m=-n}^{n}V_{nm}\begin{bmatrix}Y_{n}^m(\theta ,\phi )\end{bmatrix} \end{aligned}$$

Here, dependency of *V* on (*R*, *t*) is omitted for notation simplicity. The sensor array order $N_{a}$ is dependent on spatial sampling scheme. Assuming nearly uniform sampling scheme with zero or negligible aliasing error, the number of sensors *I* should be at least $\kappa (N_{a} + 1)^2$, where $\kappa$ assumes value in [1, 1.5]^26,27^. Equivalently, the order up to which the EEG potential can be recorded without aliasing is limited by number of sensors as

25 $$\begin{aligned} N_{a} \le \sqrt{I/ \kappa }-1 \end{aligned}$$

It is to note that (26) presents an upper limit on array order that is function of number of sensors and the value of $\kappa$. The mathematical formulation of $\kappa$ is beyond the scope of the present work.

The real valued spherical harmonics basis functions $Y_{n}^m(\theta ,\phi )$ of order *n* and degree *m* is defined as

26 $$\begin{aligned} {Y_n^m(\theta ,\phi ) = \left\{ \begin{array}{lll} (-1)^{|m|} \sqrt{2} K_n^m \sin (|m|\phi )P_n^{|m|} (\cos \theta ) &{} : m < 0 \\ (-1)^{|m|}\sqrt{2} K_n^m \cos (m\phi )P_n^m(\cos \theta ) &{} : m > 0 \\ K_n^0 P_n^0 (\cos \theta ) &{} : m=0 \end{array} \right. } \end{aligned}$$

where, the normalization constant $K_n^m$ is given by

27 $$\begin{aligned} K_n^m = \sqrt{\frac{(2n+1)(n-\mid {m}\mid )!}{4\pi (n+\mid {m}\mid )!}} \end{aligned}$$

### Spherical harmonics decomposition of EEG signal

In this Section, spatio-temporal forward model in (12) is reformulated in computationally efficient spherical harmonics domain. In practice, the continuous potential on the scalp surface is spatially sampled by placing the EEG electrodes as per the international electrode placement system. Hence, rewriting the forward SFT in (25) by replacing the integral, we have

28 $$\begin{aligned} V_{nm}&\approx \sum _{i=1}^{I} \alpha _{i}^{nm} V(\theta _{i},\phi _{i}) \end{aligned}$$

where the quadrature weights $\alpha _{i}^{nm}$ are chosen such that the approximation error involved in (29) is minimized. Substituting for $V(\theta _{i},\phi _{i})$ from inverse SFT (25) in (29), we have

29 $$\begin{aligned} V_{nm}&\approx \sum _{i=1}^{I} \alpha _{i}^{nm} \sum _{n^{'}=0}^{N_{a}}~\sum _{m^{'}=-n^{'}}^{n^{'}}V_{{n^{'}}{m^{'}}} \begin{bmatrix}Y_{n^{'}}^{m^{'}}(\theta _i,\phi _i)\end{bmatrix} \end{aligned}$$

To ensure error-free sampling, following orthonormality condition must be satisfied.

30 $$\begin{aligned} \sum _{i = 1}^{I} \alpha _{i}^{nm} Y_{n^{'}}^{m^{'}}(\theta _i,\phi _i) = \delta _{nn^{'}} \delta _{mm^{'}} \end{aligned}$$

Here $\delta$ denotes the Kronecker delta function. (31) can be rewritten in matrix form as

31 $$\begin{aligned} \begin{bmatrix} {\mathbf {Q}}\end{bmatrix}_{\Lambda _{a} \times I}\begin{bmatrix} \mathbf {Y_H}({\Phi })\end{bmatrix}_{I \times \Lambda _{a}} = \begin{bmatrix}{\mathbf {I}} \end{bmatrix}_{\Lambda _{a} \times \Lambda _{a}},~~~~~\text {where}~~~ \end{aligned}$$

32 $$\begin{aligned} \mathbf {Y_H}(\Phi ) =&\begin{bmatrix} \mathbf {Y_H}(\Omega _1)^{T}&\mathbf {Y_H}(\Omega _2)^{T}&\cdots&\mathbf {Y_H}(\Omega _I)^{T}\end{bmatrix}^{T}_{I \times \Lambda _{a}} \end{aligned}$$

33 $$\begin{aligned} \mathbf {Y_H}(\Omega _i) =&\begin{bmatrix}Y^{0}_{0}(\Omega _i)~~Y^{-1}_{1}(\Omega _i)~~Y^{0}_{1}(\Omega _i)\cdots Y^{N_{a}}_{N_{a}}(\Omega _i)\end{bmatrix} \end{aligned}$$

and $\Lambda _{a} = (N_{a}+1)^2$. The quadrature weight matrix ${\mathbf {Q}}$ is estimated in the weighted least squares sense, given by

34 $$\begin{aligned} {\mathbf {Q}} = (\mathbf {Y_H}({\Phi })^{{\mathbf {T}}} {\mathbf {W}} \mathbf {Y_H}({\Phi }))^{-1}~\mathbf {Y_H}({\Phi })^{{\mathbf {T}}} {\mathbf {W}} \end{aligned}$$

A common choice for the weighting matrix ${\mathbf {W}}$ is an identity matrix. An approximated spherical fourier transform in (30) can now be written in matrix from as

35 $$\begin{aligned} \begin{bmatrix}\mathbf {V_{SH}}\end{bmatrix}_{\Lambda _{a} \times N_s} = \begin{bmatrix} {\mathbf {Q}} \end{bmatrix}_{\Lambda _{a} \times I}\begin{bmatrix} {\mathbf {V}} \end{bmatrix}_{I \times N_s} \end{aligned}$$

and the corresponding inverse SFT is given in matrix form as

36 $$\begin{aligned} \begin{bmatrix} {\mathbf {V}} \end{bmatrix}_{I \times N_s} = \begin{bmatrix} \mathbf {Y_H} (\Phi ) \end{bmatrix}_{I \times \Lambda _{a}} \begin{bmatrix} \mathbf {V_{SH}} \end{bmatrix}_{\Lambda _{a} \times N_s} \end{aligned}$$

The quadrature weight matrix transforms the spatial domain forward data model to spherical harmonics domain data model. Multiplying ${\mathbf {Q}}$ from left to (12) results in SH domain data model as

37 $$\begin{aligned} \begin{bmatrix}{\mathbf {Q}}\end{bmatrix}\begin{bmatrix}{\mathbf {V}}\end{bmatrix} \nonumber&= \begin{bmatrix}{\mathbf {Q}}\end{bmatrix}\begin{bmatrix}{\mathbf {A}} {\mathbf {S}}\end{bmatrix} + \begin{bmatrix}{\mathbf {Q}}\end{bmatrix}\begin{bmatrix}{\mathbf {Z}}\end{bmatrix} \nonumber \\ \mathbf {V_{SH}}&= \mathbf {A_{SH}} {\mathbf {S}} + \mathbf {Z_{SH}} \end{aligned}$$

where the SH domain manifold matrix is written using (21) as $\mathbf {A_{SH}} = {\mathbf {Q}}{\mathbf {Y}}(\Phi ) {\mathbf {B}} {\mathbf {Y}}(\Psi )$. It is to be noted that the array manifold matrix of dimension *I* in spatio-temporal domain is reduced to dimension $\Lambda _{a}$, where $\Lambda _a \le I$. This reduction in dimensionality of the data is responsible for reduced computational cost in the transformed domain.

## Four shell based EEG forward model in head harmonics domain

In this Section, four shell based EEG forward model is presented in head harmonics domain. The head harmonic basis functions is first introduced followed by a detailed derivation of EEG signal decomposition in H$^2$ domain.

### The head harmonics basis function

The concept of spherical harmonics has been extended to develop a set of HemiSpherical Harmonics (HSH) basis functions that are defined over the unit hemisphere^28^. HSH basis functions have been found useful for representation of surface reflectance functions^29^, brain source localization^30^ and surface description^31^. The HSH basis functions considers the EEG potential field to be defined on the hemisphere and therefore the human head is modeled as hemisphere^30^. However, the EEG signal acquired over head assumes the shape between hemisphere and sphere. Hence, based on realistic head dimension and EEG sensor placement system, another set of basis functions (called Head Harmonics (H$^2$)) was proposed in^23,24^. It is to note that for EEG acquisition, the electrodes are placed over the head surface with an elevation angle in the range $\theta \in [0,2\pi /3]$ range^27^. Therefore, it is more appropriate to utilize the H$^2$ basis functions (where $\theta \in [0,2\pi / 3]$) instead of SH basis functions (where $\theta \in [0,2\pi ]$). The real valued orthonormal H$^2$ basis functions ($H_n^m$) are expressed as^23^

38 $$\begin{aligned} { H_n^m(\theta ,\phi ) = \left\{ \begin{array}{lll} (-1)^{|m|}\sqrt{2} {\widetilde{K}}_{n}^{m} \sin (|m|\phi )\widetilde{P}_{n}^{|m|} (\cos \theta ) &{} : m < 0 \\ (-1)^{|m|}\sqrt{2}{\widetilde{K}}_{n}^{m} \cos (m\phi ){\widetilde{P}}_{n}^{m}(\cos \theta ) &{} : m > 0\\ {\widetilde{K}}_{n}^{0}{\widetilde{P}}_{n}^{0}(\cos \theta ) &{} : m=0 \end{array} \right. } \end{aligned}$$

where ${\widetilde{K}}_{n}^{m}$ is a normalization constant and is given by

39 $$\begin{aligned} {\widetilde{K}}_{n}^{m} = \sqrt{\frac{(2n+1)(n-\mid {m}\mid )!}{3\pi (n+\mid {m}\mid )!}}. \end{aligned}$$

and ${\widetilde{P}}_{n}^{m}(x) = P_{n}^{m}(1.33x-0.33)$ is the shifted ALPs.

### Head harmonics decomposition of EEG signal

The spatio-temporal forward model in (12), is reformulated in head harmonics domain. The SH decomposition of EEG signal in (38), assumes the potential field to be sampled on the sphere. However, the anatomical brain structure and the EEG sensor array placement suggest for the potential belonging to two third (i.e $120^\circ$) of the sphere only. Hence, the data model in (38) is modified by a conversion matrix ($\beta$) that transforms the SH domain data model to H$^2$ domain. This is accomplished by changing the basis function as

40 $$\begin{aligned} H_n^m(\theta _{i},\phi _{i}) = \sum _{n'=0}^{n'=N_{a}} ~\sum _{m'=-n'}^{m'=n'}~{\beta }_{nn'}^{mm'}~Y_{n'}^{m'}(\theta _i,\phi _i) \end{aligned}$$

where the understanding of forward SFT may be used to write the element of the basis conversion matrix as

41 $$\begin{aligned} {\beta }_{nn'}^{mm'} = \int _{0}^{2\pi }\int _{0}^{{2\pi }/{3}}H_n^m(\theta ,\phi )Y_{n'}^{m'}(\theta ,\phi ) \sin \theta d\theta d\phi \end{aligned}$$

Rewriting (41) in matrix form we have,

42 $$\begin{aligned} \begin{bmatrix} {\mathbf {H}}(\Phi ) \end{bmatrix}_{I \times \Lambda _{a}} = \begin{bmatrix} \mathbf {Y_H} (\Phi ) \end{bmatrix}_{I \times \Lambda _{a}} \begin{bmatrix} {\varvec{\beta }} \end{bmatrix}_{\Lambda _{a} \times \Lambda _{a}} ,~~~~\text {where}~~~ \end{aligned}$$

43 $$\begin{aligned} {\mathbf {H}}(\Phi ) =&\begin{bmatrix} {\mathbf {H}}(\Omega _1)^{T}&{\mathbf {H}}(\Omega _2)^{T}&\cdots&{\mathbf {H}}(\Omega _I)^{T}\end{bmatrix}^{T} \end{aligned}$$

44 $$\begin{aligned} {\mathbf {H}}(\Omega _i) =&\begin{bmatrix}H^{-1}_{1}(\Omega _i)~~H^{0}_{1}(\Omega _i)~~H^{1}_{1}(\Omega _i)\cdots H^{N_{a}}_{N_{a}}(\Omega _i)\end{bmatrix} \end{aligned}$$

Multiplying ${\mathbf {Q}}$ to (43) and then utilizing the spherical array orthogonality condition in (32), the basis conversion matrix can be written as

45 $$\begin{aligned} \begin{bmatrix} {\varvec{\beta }} \end{bmatrix}_{\Lambda _{a} \times \Lambda _{a}} = \begin{bmatrix}{\mathbf {Q}}\end{bmatrix}_{\Lambda _{a} \times I}\begin{bmatrix}{\mathbf {H}}(\Phi )\end{bmatrix}_{I \times \Lambda _{a}} \end{aligned}$$

The SH domain data model in (38) can now be transferred into the H$^2$ domain data model with multiplication of the conversion matrix ${\varvec{\beta }}$ as

46 $$\begin{aligned} \begin{bmatrix}{{\mathbf {Q}}}{{\mathbf {H}}}(\Phi ){\mathbf {Q}}\end{bmatrix}\begin{bmatrix}{\mathbf {V}}\end{bmatrix} \nonumber =&\begin{bmatrix}{{\mathbf {Q}}}{{\mathbf {H}}}(\Phi ){\mathbf {Q}}\end{bmatrix}\begin{bmatrix}{\mathbf {A}} {\mathbf {S}}\end{bmatrix} + \begin{bmatrix}{{\mathbf {Q}}}{{\mathbf {H}}}(\Phi ){\mathbf {Q}}\end{bmatrix}\begin{bmatrix}{\mathbf {Z}}\end{bmatrix} \end{aligned}$$

47 $$\begin{aligned} \mathbf {T_{H^{2}}}\begin{bmatrix}{\mathbf {V}}\end{bmatrix} =&\mathbf {T_{H^{2}}}\begin{bmatrix}{{\mathbf {A}}}{{\mathbf {S}}}\end{bmatrix} + \mathbf {T_{H^{2}}}\begin{bmatrix}{\mathbf {Z}}\end{bmatrix} \end{aligned}$$

48 $$\begin{aligned} \mathbf {V_{H^{2}}} =&\mathbf {A_{H^{2}}} {\mathbf {S}} + \mathbf {Z_{H^{2}}} \end{aligned}$$

It may be noted that the transformation matrix $\mathbf {T_{H^{2}}} = {{\mathbf {Q}}}{{\mathbf {H}}}(\Phi ){\mathbf {Q}}$ transforms the spatial domain data model to the head harmonics counterpart. Additionally, the SH transformation matrix for converting the spatial domain data model to the spherical harmonics counterpart is $\mathbf {T_{SH}}=\mathbf {Q{Y_{H}}}(\Phi ){\mathbf {Q}}={\mathbf {Q}}$. In case of no transformation from the original spatial domain, the spatial transformation matrix $\mathbf {T_{Spatial}}={\mathbf {I}}$. The transformation matrices $\mathbf {T_{SH}}$ and $\mathbf {T_{H^2}}$, as detailed in Section ‘Head Harmonics Decomposition of EEG Signal’, converts the spatio-temporal data model to SH and H$^2$ domain data model, respectively. In this process, the dimensionality of the data model is changed from *I* to $\Lambda _a=(N_{a}+1)^2$, where $\Lambda _a \le I$^26,27^. This reduction in dimensionality of the data is responsible for reduced computational cost in the transformed domain.

## Spatial, SH and H^2^ based inverse brain source localization algorithms

Following the development of four shell forward data model in $\chi \in \big \{\mathbf {Spatial,~SH,~H^2}\big \}$ domain, dipole fitting algorithms are presented herein for EEG inverse problem. In particular, subspace based MUSIC^16^ and RAP-MUSIC^17^ methods are formulated in the $\chi$ domain. The subspace based methods utilize the estimated noise or signal subspace to compute the corresponding cost functions. A peak in the cost function is attributed to the neural activity. It is to note that spatial MUSIC and RAP-MUSIC algorithms utilize the spatial domain data model in (12) while the proposed SH and H$^2$ based methods utilize the data model in (38) and (48) respectively. The spatial domain EEG signal $$\begin{bmatrix}{\mathbf {V}}\end{bmatrix}$$ is first transformed to the desired $\chi$ domain counterpart by multiplying the corresponding transformation matrix ${\mathbf {T}}_{\chi }$ to obtain $$\begin{bmatrix}{\mathbf {V}}_{\chi }\end{bmatrix} = {\mathbf {T}}_{\chi }{\mathbf {V}}$$.

In the MUSIC algorithm, the estimates of active brain source location are obtained by projecting the $\chi$ domain array manifold vector $${\mathbf {a}}_{\chi }({\mathbf {r}}_p)=\begin{bmatrix}{\mathbf {T}}_{\chi }{\mathbf {a}}({\mathbf {r}}_p)\end{bmatrix}$$ over the estimated noise subspace. The $\chi$ domain MUSIC spectrum is expressed as

49 $$\begin{aligned} {{\mathbf {J}}_{\chi }({\mathbf {r}}_p,{\mathbf {e}}_p)_{\text {MUSIC}}} = \frac{\begin{bmatrix}{\mathbf {a}}_{\chi }^{{\mathbf {T}}} ({\mathbf {r}}_p)\end{bmatrix}\begin{bmatrix}{\mathbf {a}}_{\chi } ({\mathbf {r}}_p)\end{bmatrix}}{\begin{bmatrix}{\mathbf {a}}_{\chi }^{\mathbf {T}} ({\mathbf {r}}_p)\end{bmatrix} \begin{bmatrix}{\mathbf {P}_{\chi }}\end{bmatrix}\begin{bmatrix}{\mathbf {a}}_{\chi } ({\mathbf {r}}_p)\end{bmatrix}}, \end{aligned}$$

where ${\mathbf {P}}_{\chi }$ is the noise subspace projection matrix obtained from eigen value decomposition of covariance matrix $${\mathbf {R}}_{\chi }= E\begin{bmatrix}{\mathbf {V}}_{\chi }{\mathbf {V}}_{\chi }^{{\mathbf {T}}}\end{bmatrix}$$^16^. The MUSIC spectrum in (49) can also be written in a simplified manner as^23^

50 $$\begin{aligned} {{\mathbf {J}}_{\chi }({\mathbf {r}}_p,{\mathbf {e}}_p)_{\text {MUSIC}}} = \frac{1}{\lambda _{min}(\begin{bmatrix}{\mathbf{U}}_{\chi }^{{\mathbf {T}}}\end{bmatrix}\begin{bmatrix}{{\mathbf {P}}_{\chi }}\end{bmatrix}\begin{bmatrix}{\mathbf{U}}_{\chi }\end{bmatrix})} \end{aligned}$$

where $\lambda _{min}(.)$ is the minimum eigenvalue of (.), and ${{\mathbf {U}}}_{\chi }$ is the Left Singular Vector (LSV) of ${\mathbf {T}}_{\chi }G({\mathbf {r}}_p)$. One of the drawback of MUSIC algorithm is that it searches for multiple local peaks in the entire head volume, which makes the task time consuming and susceptible to false source localization. To overcome the limitation, RAP-MUSIC was proposed that extracts the global maxima at each recursion steps. For the *k*th recursion step, the $\chi$ domain RAP-MUSIC spectrum is defined as

51 $$\begin{aligned} {{\mathbf {J}}_{\chi }^{k}({\mathbf {r}}_p,{\mathbf {e}}_p)_{\text {RAP-MUSIC}}} = \frac{1}{\lambda _{min}(\begin{bmatrix}{{\mathbf {U}}}_{\chi }^{k {\mathbf {T}}}\end{bmatrix}\begin{bmatrix}{{\mathbf {P}}_{\chi }^{k}}\end{bmatrix}\begin{bmatrix}{\mathbf{U}}_{\chi }^{k}\end{bmatrix})} \end{aligned}$$

where ${{\mathbf {U}}}_{\chi }^{k}$ is the LSV of $$\begin{bmatrix}\Uppi ^{\bot }_{\mathbf {{\widetilde{A}}}_{k-1}} {\mathbf {T}}_{\chi }~ G({\mathbf {r}}_p)\end{bmatrix}$$ and $${{\mathbf {P}}_{\chi }^{k}} = \begin{bmatrix}\Uppi ^{\bot }_{\mathbf {{\widetilde{A}}}_{k-1}}{{\mathbf {P}}_{\chi }}\end{bmatrix}$$. The orthogonal projector $\Uppi ^{\bot }_{\mathbf {{\widetilde{A}}}_{k-1}}$ projects out the topography of the already found sources as

52 $$\begin{aligned} \Uppi ^{\bot }_{\mathbf {{\widetilde{A}}}_{k-1}} = {\mathbf {I}} - {\mathbf {{\widetilde{A}}}_{k-1}}(\mathbf {\widetilde{A}}_{k-1}^{{\mathbf {T}}}\mathbf {{\widetilde{A}}}_{k-1})^{-1}{\mathbf {{\widetilde{A}}}_{k-1}^{{\mathbf {T}}}} \end{aligned}$$

with $\Uppi ^{\bot }_{\mathbf {{\widetilde{A}}}_{0}} = {\mathbf {I}}$. Matrix $\mathbf {{\widetilde{A}}}_{k-1}$ concatenates the array manifold vectors of already found sources, and is given by

53 $$\begin{aligned} \mathbf {{\widetilde{A}}}_{k-1} = \begin{bmatrix} \big \{{{\mathbf{T}}_{\chi }{{\mathbf {a}}}}({\mathbf {r}}_1)\big \}&\cdots&\big \{{{{\mathbf {T}}_{\chi }}{{\mathbf {a}}}}(\mathbf {r}_{k-1})\big \}\end{bmatrix} \end{aligned}$$

## Simulated data analysis

Various experiments were conducted to illustrate the advantages of SH and H$^2$ domain based BSL algorithms. Both simulated and real EEG data were utilized for this purpose. In simulation, four layer concentric shell head model with radius of brain, CSF, skull, and scalp was considered to be 8.0 *cm*, 8.2 *cm*, 8.7 *cm*, and 9.2 *cm* respectively. The brain and scalp conductivities were set to 0.33 $(\Omega m)^{-1}$. The conductivities of skull and CSFs were taken to be 1/80 and 5 times of that of the brain. A total of $I = 128$ Sensors were placed over head scalp utilizing $10-5\%$ electrode placement system. The order $N_{ref}$ was set to 60 considering the diminishing nature of EEG mode strength for higher order. The number of discrete time samples $N_S$ was 200. The inter-grid gap was chosen to be 1 *mm*. The true source grid (i.e., the Region Of Interest (ROI)) was a 2-D axial slice at the depth of 3.2 *cm* from the top of the scalp surface as shown in Fig. 2b. The simulated sources were placed pseudo-randomly on ROI so that they were at least 3 *cm* from each other and at least 2 *cm* away from the center of ROI. Example of such a source distribution for $P=4$ is shown in Fig. 2c. Active source location and orientation is assumed to be fixed. Potential data were generated considering the radial orientation of active brain sources. All experiments were conducted with $L = 200$ Monte-Carlo repetitions. It may be noted that the peak of the spectrum was explored only at ROI to reduce the computation load of the search over the entire volume.

**Figure 3:**
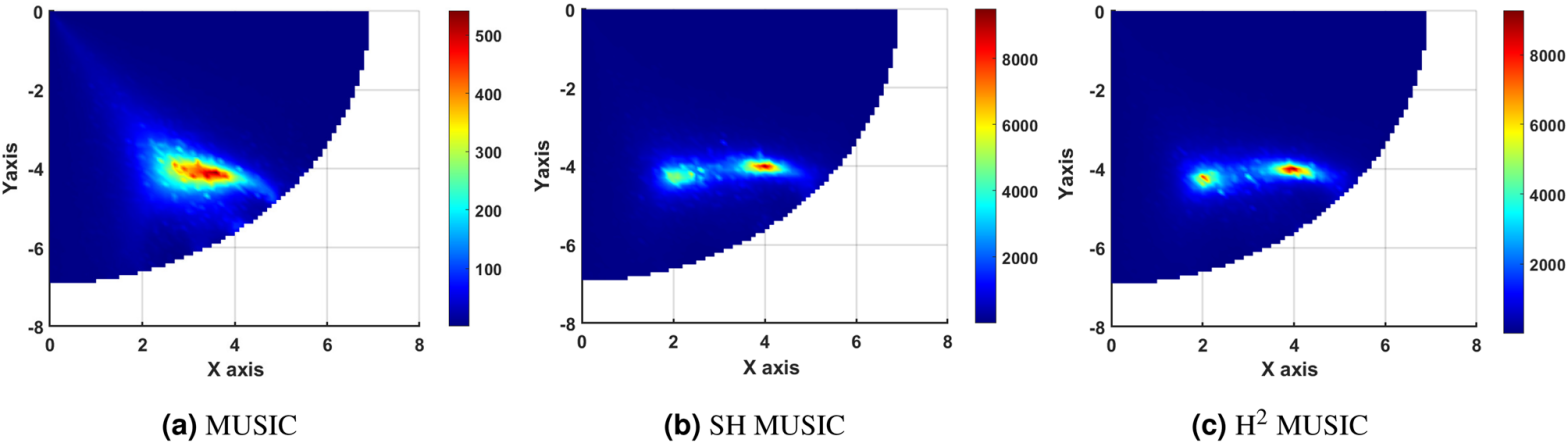
3D view of (**a**) spatial MUSIC, (**b**) SH MUSIC and (**c**) H$^2$ MUSIC for two active dipole source.

### Spatial resolution

The capability of BSL algorithms in estimating the spatially closed active source location is analysed herein by plotting the respective cost function spectrum. Two dipole sources were placed at $(2cm, -4cm, 6cm)$ and $(4cm, -4cm, 6cm)$ on ROI. The inter source correlation and distance between the two sources was taken to be 0.2 and 2 *cm* respectively. The Signal to Noise Ratio (SNR) was set to 5*dB*. The order $N_{a}$ was taken to be 3 for both SH and H$^2$ domain. The 3-D view of spatial MUSIC, SH-MUSIC and H$^2$-MUSIC cost function is plotted in Fig. 3 (a)-(c). It may be noted that the spatial MUSIC fails to localize closely spaced active dipoles at low SNR, providing a single false location. However, the proposed SH and H$^2$ counterparts localizes the two sources well with high resolution. This may be attributed to the increased contribution of the initial two eigenvalues of the covariance matrix corresponding to the two sources. The contribution of the initial two eigen values is $80.61\%$ in spatial domain. This contribution increases to $98.55\%$ and $98.62\%$ in the SH and H$^2$ domain respectively.

**Figure 4:**
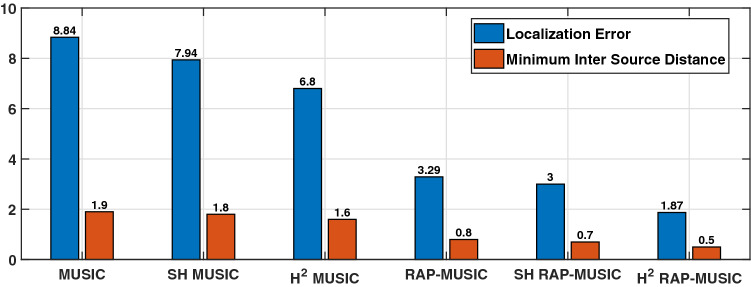
Spatial resolution.

Additionally, the minimum distance between the two sources that have been localized via different BSL algorithms are quantified. Two sources were placed pseudo-randomly on ROI so that they were at least 2 cm away from origin. The inter source distance was varied from 0.5 cm to 2.0 cm. The minimum inter source distance is the shortest possible distance between two sources that can be localized. The minimum inter source distance and the corresponding localization error is illustrated in Fig. 4. It is to note that H$^2$ based MUSIC and RAP-MUSIC attains least localization error and minimum inter source distance when compared to spatial and SH counterparts. In particular, the H$^2$ RAP-MUSIC has the best performance.

**Table 1:** LE of (**a**) MUSIC, (**b**) SH MUSIC, and (**c**) H$^2$ MUSIC with different SNR values and array order $N_{a}$.

$N_a$	SNR														
	-5 dB			0 dB			5 dB			10 dB			15 dB		
	(a)	(b)	(c)	(a)	(b)	(c)	(a)	(b)	(c)	(a)	(b)	(c)	(a)	(b)	(c)
**1**	11.702	51.891	44.653	5.123	36.770	37.933	1.606	44.382	44.405	0.557	37.388	36.441	0.097	34.258	37.006
**2**		7.141	6.379		3.311	2.936		1.028	0.738		0.177	0.147		0.044	0.014
**3**		8.175	7.995		3.052	3.231		0.930	0.885		0.128	0.123		0.024	0.024
**4**		9.841	9.722		3.705	3.614		1.073	1.133		0.244	0.244		0.048	0.038
**5**		10.224	10.350		4.168	4.093		1.274	1.280		0.373	0.367		0.062	0.80
**6**		11.109	10.823		4.426	4.394		1.424	1.300		0.415	0.379		0.082	0.062
**7**		11.528	11.435		4.644	4.631		1.448	1.447		0.514	0.433		0.072	0.082
**8**		11.690	11.569		4.833	4.825		1.535	1.469		0.503	0.513		0.095	0.082
**9**		12.033	11.708		5.018	4.950		1.592	1.550		0.513	0.523		0.111	0.115
**10**		12.246	12.158		5.161	5.022		1.613	1.574		0.537	0.619		0.127	0.121

### Localization error

In this Section, the Localization Error (LE) of spatial, SH and H$^2$ based BSL methods is presented. The LE for *L* iteration and *P* dipole source is defined as

54 $$\begin{aligned} \text{ LE } = \frac{1}{L}\sum _{l = 1}^{L} \sqrt{\frac{1}{P} {\sum _{p=1}^{P}{(x_{p}- {\widetilde{x}}_{pl})^{2}}+{(y_{p}- \widetilde{y}_{pl})^{2}} }} \end{aligned}$$

where (*x*, *y*) is the true source location and $({\widetilde{x}}, {\widetilde{y}})$ is the estimated source location. Simulation experiments were conducted to compute the LE with varying SNRs, array orders, number of active dipoles, and inter source correlation.

#### LE with varying SNR and array order for single source

The number of active dipole is taken to be $P=1$ to study the effect of varying SNR and array order on LE. The source was placed in the pseudo-random manner. The LE is presented in Table 1 for MUSIC method in spatial, SH and H$^2$ domain. As expected, LE decreases consistently with increasing SNR. LE variation is additionally presented with varying array order $N_{a} \in \{1,\ldots ,10\}$ in accordance with (26). High localization error of SH and H$^2$ MUSIC methods was observed for array order $N_{a}=1$. This can be attributed to poor signal representation at lower order. The error further decreases with increase in order to give an optimum array order for a given number of sources. It is interesting to note that for single source, array order $N_{a}=\{2,3\}$ has the least localization error as highlighted in the Table 1 for SH/H$^2$ based localization. An increase in the localization error was observed thereafter thus giving an upper bound on the array order. This is consistent with the array signal processing^20^. In general, it may be observed that the H$^2$ MUSIC method outperforms the SH counterpart. For multiple source localization, the optimal array order with the least localization error is presented next.

**Figure 5:**
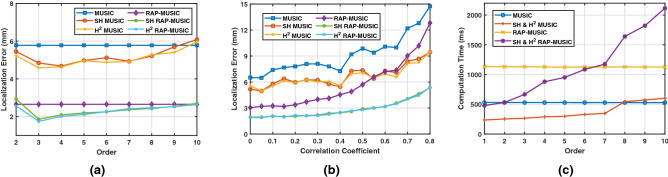
Performance of Spatial, SH and H$^2$ based MUSIC, RAP-MUSIC for two concurrent active sources at 5 dB SNR, (**a**) Localization error with varying array order $N_{a}$ (**b**) Localization error with varying inter source correlation (**c**) Computation time with varying array order $N_{a}$.

#### LE with varying array order for two sources

Performance of spatial, SH and H$^2$ based MUSIC only method was explored in previous Section. In this Section, performance of MUSIC and RAP-MUSIC is presented for two active dipole sources with inter-source correlation as 0.2, placed pseudo randomly at 5 dB SNR. Localization error of MUSIC and RAP-MUSIC methods in the three domain with varying order $N_a$ is presented in Fig. 5a. As the spatial methods are independent of the array order, the localization error remains constant with order $N_a$. It may be noted that H$^2$, SH domain methods outperformed spatial counterparts. A valley in localization error plot may be observed at order 3 for the two active dipole sources. In all the three domains, the performance of RAP-MUSIC is better than MUSIC.

#### LE analysis for RAP-MUSIC with varying array order and number of active dipoles

Optimum array order is experimentally estimated in this Section with varying number of active dipole sources. With an increase in the number of active sources, finding the local maxima in the MUSIC spectrum is susceptible to false source localization. Hence, LE results are presented in Table 2 for RAP MUSIC method only. The number of sources were varied from 1 to 5 at 5 dB SNR. The inter-source correlation between each adjacent pair was set to [0.20, 0.17, 0.17, 0.20]. It may be noted that the spatial domain RAP-MUSIC method localizes sources upto 3. On the other hand, the proposed SH and H$^2$ methods successively localize all the five sources. An upward shift in optimal array order with an increase in the number of active sources was observed. This may be because of $\kappa$ that appears to be dependent on the number of active dipole sources. Variation in the optimum array order with varying number of sources is highlighted in Table 2.

#### LE with varying inter source correlation

The subspace based source localization methods work on the prior assumption that the active sources are uncorrelated. When inter source correlation is significant (synchronous source case), BSL methods provide erroneous estimation of dipole sources. Therefore, to study the applicability of SH and H$^2$ based methods in the realistic scenario of synchronous source activation is of great significance. In the present simulation, the pearson correlation coefficient was varied from 0 to 0.8 for the case of two simultaneously active dipole sources at 5 dB SNR. The harmonics order for the SH and H$^2$ based methods was taken to be 3 as discussed in Fig. 5b. Effect of inter source correlation on the localization error is presented in Fig. 5b. It can be observed that the SH and H$^2$ domain based methods perform relatively better than their spatial domain counterparts in synchronous source localization. In particular the H$^2$ based method outperforms the rest.

**﻿Table 2:** Localization error analysis of RAP-MUSIC in different domain with varying number of sources.

$N_a$	Domain	Number of Sources				
		1	2	3	4	5
N.A.	Spatial	1.606	2.650	6.247	-	-
2	SH	1.028	2.931	6.096	12.892	24.390
	H$^2$	0.738	2.562	4.949	12.126	23.441
3	SH	0.930	1.860	2.426	3.421	4.363
	H$^2$	0.885	1.744	2.348	3.466	4.585
4	SH	1.073	2.089	2.443	3.119	3.805
	H$^2$	1.133	2.008	2.393	3.120	3.798
5	SH	1.274	2.188	2.604	3.154	3.846
	H$^2$	1.280	2.107	2.575	3.224	3.851
6	SH	1.424	2.253	2.721	3.450	3.947
	H$^2$	1.300	2.264	2.688	3.301	3.902
7	SH	1.448	2.367	2.873	3.624	4.195
	H$^2$	1.447	2.419	2.837	3.570	4.174

**Figure 6:**
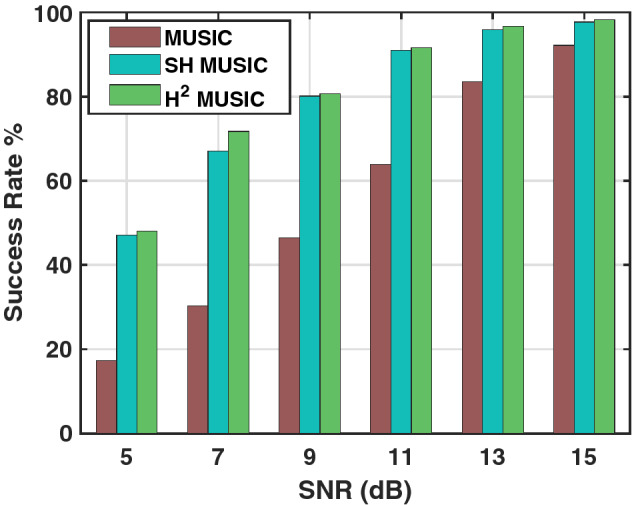
Success rate $\%$ of MUSIC in the spatial, SH and H$^2$ domain.

### Computation time

The computational efficiency of SH and H$^2$ domain based BSL methods such as MUSIC and RAP-MUSIC is presented herein. The transformation matrices $\mathbf {T_{SH}}$ and $\mathbf {T_{H^2}}$, as detailed in Section ‘Head Harmonics Decomposition of EEG Signal’, converts the spatio-temporal data model to SH and H$^2$ domain, respectively. In this process, the dimensionality of the data model is changed from *I* to $\Lambda _a=(N_{a}+1)^2$, where $\Lambda _a \le I$^26,27^. This reduction in dimensionality of the data is responsible for reduced computational cost in the transformed domain. The advantage of data model formulation in SH and H$^2$ domain in terms of dimensionality reduction is illustrated as a reduction in computation time. A 64-bit type system having Intel Core i7-6700 processor with 4 cores @ 3.40 GHz, and 16 GB RAM was utilized. Windows 10 Pro Version 1803 with MATLAB Version: R2019b was used for the evaluation. Computation time was measured using tic, toc MATLAB function at 5dB SNR. In the simulation, number of dipole sources were taken to be $P=2$. The average computation time was evaluated with varying sensor array order $N_{a} \in \{1,\ldots ,10\}$, as presented in Fig. 5c. It was observed that the computation time for SH and H$^2$ based methods is same. Present simulation demonstrate the effectiveness of SH and H$^2$ domain methods when compared with spatial domain methods. A significant reduction in the computation time for harmonics order $N_{a} \in [1,5]$ is observed. It may be noted from Sect. 7.2 that the optimum harmonic order for two sources is 3. The high computational cost involved in solving the inverse problem using conventional spatial domain methods is a major obstacle in real-time EEG source localization.

### Success rate

At a particular SNR value, the success rate of an algorithm is defined as the ratio of the number of localizations achieved with a 0 localization error to the total number of attempts. We further extend our performance evaluation by examining the success rate in $\%$ for spatial, SH and H$^2$ domain MUSIC methods. The number of Monte-Carlo trials were taken to be L = 1000. The order of SH and H$^2$ harmonics was set to 2. The success rate in $\%$ for a single source is presented in Fig. 6. At low SNR, significant difference in success rate can be observed between the SH, H$^2$ and spatial domain methods. In particular, the H$^2$ MUSIC is seen as the robust and most accurate source localization algorithm.

## Real data analysis

For epilepsy surgery, localization of the seizure focus is time consuming and requires evaluation of neurological tests by experienced clinicians. In this Section, additional validation of the proposed head harmonic-based BSL is presented for epileptic seizure location using real clinical EEG data.

### Subjects

An electrophysiological and imaging data of five patients with drug resistant epilepsy of long duration were included for study. The mean age of the cohort was 24.4 ± 7.33 with standard deviation range as 15 to 34 year. The patients were admitted to the Deenanath Mangeshkar Hospital & Research Center at Pune, Maharashtra, India. All of these patients were on multiple Anti-Seizure Medications (ASM). All patients underwent thorough pre-surgical evaluation with long term Video EEG, 3 Tesla Magnetic Resonance Imaging (MRI) scan, Positron Emission Tomography (PET) scan and neuropsychological assessments. All the patients had lesion on the MRI along with corresponding hypo metabolism on the PET scan with good electrophysiological concordance. All of these patients were discussed in weekly multidisciplinary meetings and were advised surgical resection. Imaging data was blinded and researchers did not know the site or side of the lesion before EEG source analysis. Informed Consent is obtained from all subjects and the study is conducted in accordance with relevant guidelines and regulations approved by the institutional ethical committee of Deenanath Mangeshkar Hospital & Research Centre (Ref: DMHRC Code- IHR/2021/Apr/NK/403).

### EEG recordings

EEG recordings were done using standard 10-20 international system (19 electrodes along with ground and reference electrodes). Additional two electrodes T1 and T2 were added to detect anterior temporal discharges along with Electromyography (EMG) and Electrocardiography (ECG) electrodes. All the electrodes were pasted using standard gel and impedance was kept below 5k$\Omega$. Sampling frequency of EEG was set 2 KHz. Signals were passed through band pass filter of 1 Hz -70 Hz, followed by Notch filter to remove ’50Hz’ power line interference during recording. An epoch of 20 seconds was selected for source analysis by expert lab technician from the long duration recorded EEG, where first five seconds were pre-ictal recordings and another 15 seconds were seizure onset and propagation across the channels. Data pre-processing was performed offline on the selected epoch of data using the EEGLAB toolbox. The EMG and ECG channels present in the dataset were not considered.

**Figure 7:**
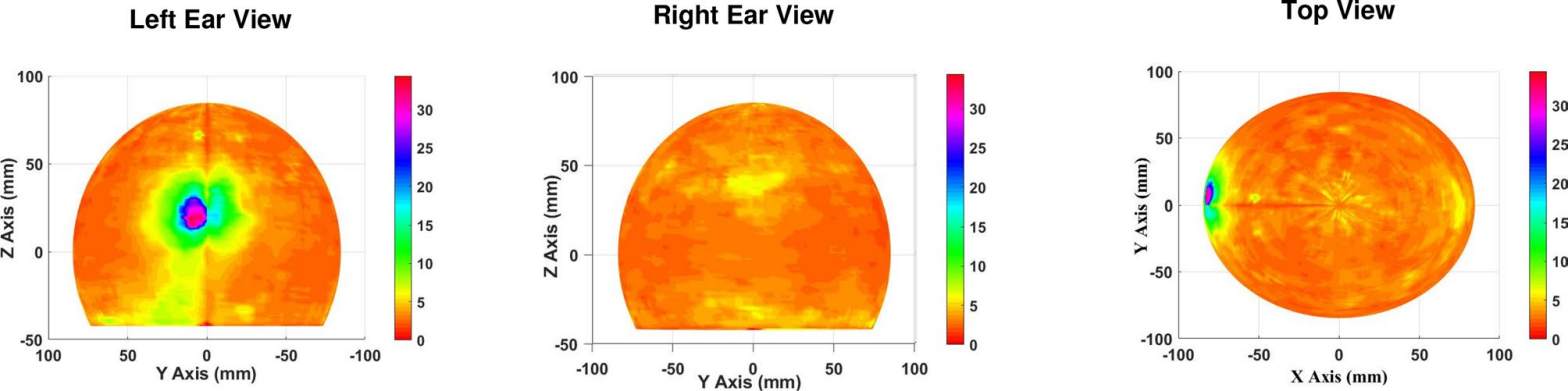
H$^2$ MUSIC cost function plot of a subject P1 having seizure on left temporal lobe.

### Source localization

Head harmonic based MUSIC algorithm is evaluated on the real epileptic seizure data as it provides all the peaks corresponding to active dipoles simultaneously. The data recorded from 21 channels is interpolated to 64 channel data using EEGLab toolbox ‘spherical’ interpolation method. The number of source was assumed to be two as eigen value decomposition of covariance matrix had 95% of its energy confined to a rank-2 subspace. Five epileptic patients data were analyzed. Epileptogenic zone was observed in the left temporal lobe for the patients P1, P2, P3 and P5. The patient P4 had epileptogenic zone in the right temporal lobe. Fig. 7 illustrates the H$^2$ MUSIC cost function plot of patient P1 having epiletogenic zone on the left temporal lobe. A pronounced cortical activation is focused in the corresponding epileptic region. The results are validated by the neurosurgeon who performed standard temporal lobe resection surgeries on left side in 4 patients (P1, P2, P3, P5) and on right side in 1 patient (P4). Our result demonstrates the effectiveness of H$^2$ domain algorithm in successfully localizing the true epileptic activation region on real EEG data.

### Ethical approval

All methods were carried out in accordance with relevant guidelines and regulations approved by the institutional ethical committee of Deenanath Mangeshkar Hospital and Research Centre (Ref: DMHRC Code- IHR_2021_Apr_NK_403 , Dated: 2nd Apr 2021). A generic patient consent is taken but specific consent is waived off by ethics committee because this study did not alter the patient management and it’s only involves studying of data acquired for other reason.

## Conclusion

In this work, we proposed Spherical Harmonics (SH) and Head Harmonics (H$^2$) domain processing framework for active dipole localization. The four shell head model in spatio-temporal domain is formulated in computationally efficient SH and H$^2$ domain. A qualitative and quantitative performance comparison of Multiple Signal Classification (MUSIC) and Recursively Applied and Projected (RAP)-MUSIC methods in spatial, SH and H$^2$ domain is presented on simulated data. SH and H$^2$ domain processing effectively solves the problem of high computational cost without sacrificing the inverse source localization accuracy. The performance of H$^2$ based RAP-MUSIC was best when compared with SH and spatial domain counterparts. The performance dependency of SH and H$^2$ domain methods on array order was experimentally established. The proposed H$^2$ MUSIC was additionally validated for epileptogenic zone localization using clinical EEG data. The proposed framework offers an effective solution for clinicians in automated and time efficient seizure localization.
